# Clinical adoption of deep learning target auto-segmentation for radiation therapy: challenges, clinical risks, and mitigation strategies

**DOI:** 10.1093/bjrai/ubae015

**Published:** 2024-10-25

**Authors:** Alessia De Biase, Nanna Maria Sijtsema, Tomas Janssen, Coen Hurkmans, Charlotte Brouwer, Peter van Ooijen

**Affiliations:** Department of Radiation Oncology, University Medical Centre Groningen (UMCG), Groningen, 9700 RB, The Netherlands; Data Science Centre in Health (DASH), University Medical Centre Groningen (UMCG), Groningen, 9700 RB, The Netherlands; Department of Radiation Oncology, University Medical Centre Groningen (UMCG), Groningen, 9700 RB, The Netherlands; Department of Radiation Oncology, The Netherlands Cancer Institute, Amsterdam, 1006 BE, The Netherlands; Department of Radiation Oncology, Catharina Hospital Eindhoven, Eindhoven, 5602 ZA, The Netherlands; Department of Electrical Engineering, Technical University Eindhoven, Eindhoven, 5600 MB, The Netherlands; Department of Radiation Oncology, University Medical Centre Groningen (UMCG), Groningen, 9700 RB, The Netherlands; Department of Radiation Oncology, University Medical Centre Groningen (UMCG), Groningen, 9700 RB, The Netherlands; Data Science Centre in Health (DASH), University Medical Centre Groningen (UMCG), Groningen, 9700 RB, The Netherlands

**Keywords:** target auto-segmentation, deep learning, risk analysis, monitoring, quality assurance, clinical adoption, radiation oncology

## Abstract

Radiation therapy is a localized cancer treatment that relies on precise delineation of the target to be treated and healthy tissues to guarantee optimal treatment effect. This step, known as contouring or segmentation, involves identifying both target volumes and organs at risk on imaging modalities like CT, PET, and MRI to guide radiation delivery. Manual segmentation, however, is time-consuming and highly subjective, despite the presence of contouring guidelines. In recent years, automated segmentation methods, particularly deep learning models, have shown promise in addressing this task. However, challenges persist in their clinical use, including the need for robust quality assurance (QA) processes and addressing clinical risks associated with the use of the models. This review examines the challenges and considerations of the clinical adoption of deep learning target auto-segmentation in radiotherapy, focused on the target volume. We discuss potential clinical risks (eg, over- and under-segmentation, automation bias, and appropriate trust), mitigation strategies (eg, human oversight, uncertainty quantification, and education of clinical professionals), and we highlight the importance of expanding QA to include geometric, dose-volume, and outcome-based performance monitoring. While deep learning target auto-segmentation offers significant potential benefits, careful attention to clinical risks and rigorous QA measures are essential for its successful integration in clinical practice.

## Setting the stage: segmentation in radiation therapy

**Figure 1.**
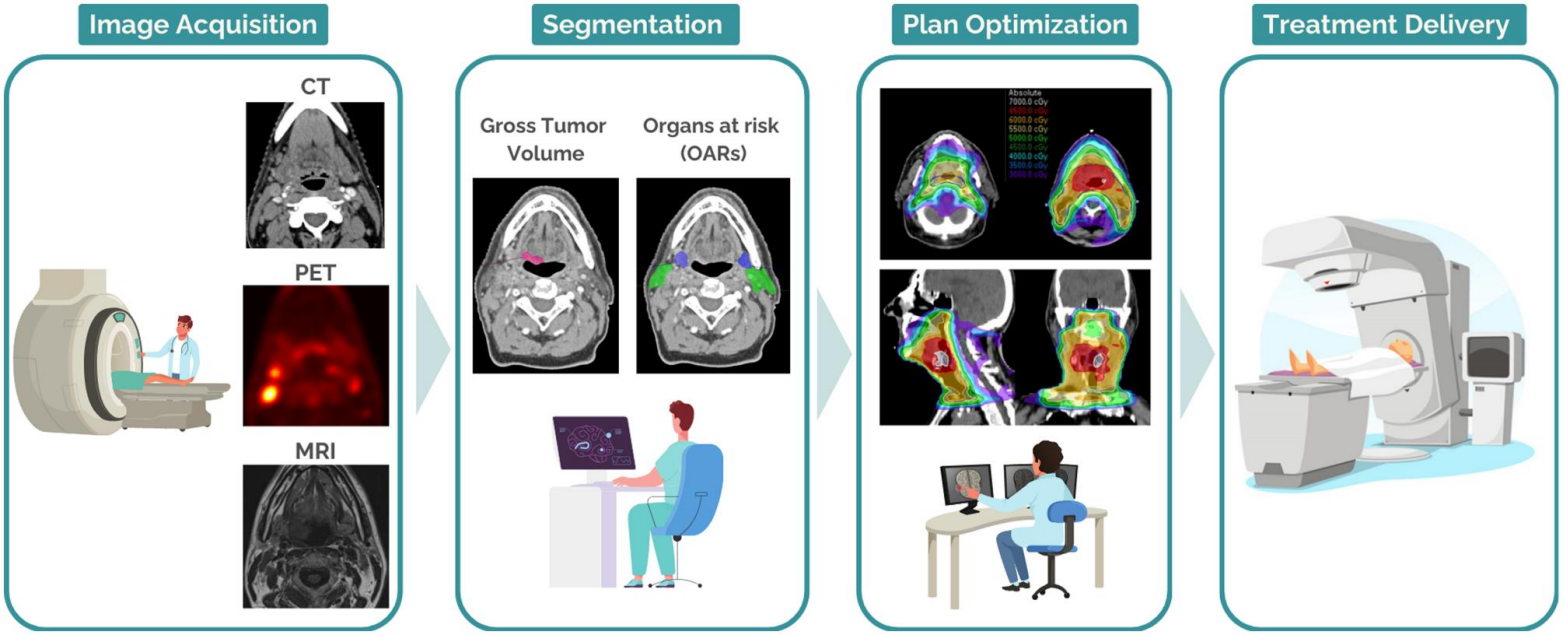
Illustration of the different steps of the radiation therapy treatment journey, showing an example of a head and neck cancer patient. In the image acquisition step, CT, PET, and MRI scans are acquired in treatment position. Subsequently, segmentations are performed for the tumour that is visible on imaging (gross tumour volume) and in clinical examinations, and the adjacent organs (organs at risk). In the third step, the radiation plan is optimized based on the patient’s CT scan and on the segmentations performed in the previous step. Finally, as the last step, the treatment is delivered.

Radiation therapy is a localized cancer treatment that employs high-energy radiation directed at a specific volume of the body to kill cancer cells. Achieving optimal treatment effect requires the exact identification of the tumour location and extension. Often, multiple 3D medical imaging modalities such as CT, PET, and MRI are combined in this process, since tumours are not always visible on single imaging modalities. These images, in combination with additional diagnostic or clinical information (tumour staging, physical examination, etc) are used by the radiation oncologist to identify the radiation target volume. The delineation of this volume is subsequently used to optimize the radiation beam fluence, aiming at delivering sufficient dose in the tumour and preserving the surrounding healthy tissues. Delineation of the target, also known as contouring or segmentation, is indispensable for achieving the therapeutic effect of the treatment (Figure 1).

In the segmentation task, two subgroups of structures can be distinguished: organs at risk (OARs), concerning all the healthy tissues/organs located in the radiation field, and the target volumes, which should receive sufficient dose to achieve local tumour control. The delineated OARs are used during treatment plan optimization to minimize radiation damage to these critical volumes, which could result in toxicity and complications. Identifying the boundaries of OARs is generally considered a less complex task compared to delineating the target volume. One significant reason is that OARs are typically anatomically defined structures or organs and therefore their location, shape, volume, and appearance generally exhibit consistent traits across patients, in contrast to tumours, where these factors can vary significantly^1^.

**Figure 2.**
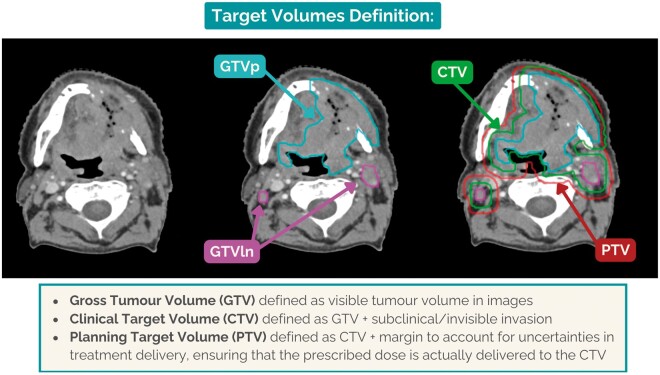
Illustration of an example of the target volumes contours on the axial slice of a CT scan used for radiotherapy planning of a head and neck cancer patient. The first image on the left shows the Planning CT only. In the middle image, the gross tumour volume of the head and neck primary tumour (GTVp) and the gross tumour volume of the pathologic lymph nodes (GTVln) are displayed. The image on the right side illustrates how the clinical target volume and planning target volume align with the GTV segmentations, in this case, offering a clear visual depiction of their interrelation with the preceding volumes. Abbreviation: GTV = gross tumour volume.

In radiotherapy, the word ‘target’ may be used to indicate different structures, as illustrated in the example in Figure 2. The definitions provided below are in accordance with the International Commission on Radiation Units and Measurements (ICRU) Report 50^2^. Gross tumour volumes (GTV) are the visible tumours in imaging and in clinical examinations, representing the demonstrable extent of the malignant growth. Clinical target volumes (CTV) are targets which account for the presence of subclinical microscopic tumour spread beyond the visible GTV. The planning target volume (PTV) is derived by adding a margin to the CTV to compensate for uncertainties in delineation, treatment delivery (eg, beam alignment, patient positioning) and patient anatomy (eg, organ motion and organ deformation). To precisely delineate the GTVs, a variety of imaging techniques is often needed. Conversely, defining CTVs involves not only imaging but also clinical information and anatomical considerations (eg, adjustments may be needed to exclude bone structures if the margin extends beyond them). The CTV is either defined based on GTV plus margin (referred to as the tumour CTV), anatomical regions (eg, prostate gland, lymph nodes levels), or a combination of both. CTV delineation guidelines vary based on tumour sites and staging^3–7^.

Over the last two decades, CT imaging has stood as the primary modality in radiotherapy treatment, providing essential information for dose calculation, since CT Hounsfield units are easily related to radiation beam attenuation. Nonetheless, CT does not always offer sufficient soft-tissue or functional contrast crucial for visualizing tumour boundaries. To overcome this limitation, pre-treatment PET and/or MRI images are often co-registered with the planning CT or utilized independently in more advanced treatment techniques (eg, MR-only radiotherapy). Alongside imaging, clinical examinations of the patient provide valuable information for exact tumour localization. The role of the radiation oncologist is to finally integrate all this complementary data to manually segment the target on the available modalities.

Interpretation of imaging by radiation oncologists is highly subjective^8^ and dependent on clinical judgement, leading to inter- and intra-observer variability in target segmentation^9–11^. These differences in how the same structure is segmented by different or even the same observer, under similar circumstances, lead to inconsistencies in the dose delivery to the actual tumour volume^12^. Since the introduction of intensity-modulated radiotherapy, which results in steeper dose gradients, the need for more standardized target volume definitions increased^13^^,^^14^. As a consequence, an increase of tumour site-specific consensus delineation guidelines was observed^15^. However, despite their implementation, significant inter-observer variability persists^16^, which may depend on the difficulty in their interpretation^17^.

In summary, target segmentation is a time-consuming and subjective task, hence prone to error and inter-observer variability, highlighting the need for solutions that can enhance efficiency, accuracy, and consistency. Moreover, with the shortage of staff and the introduction of innovative techniques like online adaptive radiotherapy, rapid target segmentation becomes crucial in ensuring timely and accurate treatment delivery. In the era of automation and artificial intelligence (AI), deep learning (DL) models, trained on large amounts of annotated data, showed promises in learning complex patterns in different data sources to automate the segmentation process^18^. However, challenges remain in the widespread adoption of automated target segmentation in clinical settings^19^.

The NRG Oncology recently published an assessment of AI-based organ auto-segmentation models for radiation therapy, evaluating their clinical utilization, accuracy, and challenges with a focus on their integration, potential benefits, and limitations^20^. However, as previously mentioned, significant differences exist between organ and target segmentation tasks. Building on this, our review aims to offer a comprehensive understanding of the unique challenges and considerations associated with the clinical adoption of target auto-segmentation technologies. Additionally, we draw attention to the crucial aspects of clinical risk analysis and monitoring to ensure the safe and effective utilization of these automated systems in radiotherapy treatment preparation. More specifically, the manuscript is organized as follows:

- The second section reviews the state-of-the-art DL models for target auto-segmentation;
- The third section examines the current landscape, challenges, and steps needed for clinical implementation and adoption of AI-based target auto-segmentation;
- The fourth section addresses the sources of failure in DL models, their clinical implications, and strategies for risk mitigation;
- The fifth section highlights the critical role of QA in radiotherapy, with a focus on DL target auto-segmentation models, and discusses challenges and strategies for optimization;
- The sixth section looks at future directions for DL target auto-segmentation in radiotherapy;
- The seventh section summarizes the key findings and discussion points of the review.

## Deep learning target auto-segmentation

Auto-segmentation techniques have a long history of usage in radiotherapy. Rong et al^20^ provided a complete overview, highlighting the advantages and disadvantages of those techniques in the clinical setting. When compared to the previously adopted methods, DL-based approaches stand out for increased robustness, and higher efficiency and performance. In fact, a significant drawback of prior methodologies lies in their heavy reliance on a single image characteristic including shape, appearance, and intensity (eg, thresholding or edge-based approaches) of structures. Moreover, commonly employed atlas-based approaches assume sufficient similarities between the atlas cases (used as a reference to guide the labelling of new data) and the new cases, enabling deformable transformations to achieve accurate new segmentations. While this is often the case for OARs, it could pose considerable challenges for target delineation, where significant variability in tumour shape, size, and location can occur.

Savjani et al^19^ and Matoska et al^21^ provided an overview of site-specific advances in GTV and CTV auto-segmentation in radiotherapy, respectively, using DL techniques. Building upon their contributions, we provide an updated version in Table 1, including the latest and best-performing target auto-segmentation studies published from 2022 to March 2024. A literature search was conducted in PubMed and in Google Scholar using the following keywords: (‘tumor’ OR ‘tumour’ OR ‘cancer’) AND (‘segmentation’ OR ‘automatic segmentation’ OR ‘automated segmentation’ OR ‘auto-segmentation’ OR ‘contouring’ OR ‘delineation’) AND (‘deep learning’). Performance was assessed using segmentation accuracy metrics, with the dice similarity coefficient (DSC) being the most commonly employed. While DSC is frequently used, it is important to note that it has limitations, especially due to its heavy reliance on volume. Other metrics were also considered when available. Multi-centre studies were preferred; however, in the absence of such studies, we included single-centre ones. Studies with the largest patient cohorts were selected. In some tumour sites (glioblastoma, oropharynx, head and neck pathologic lymph nodes, kidney, and liver), this involved including the latest and best-performing results from DL programming challenges. The previously published review was used as reference to identify studies that achieved the highest or similar performance in comparison.

**Table 1.** Overview of best performing DL target auto-contouring methods by tumour site (January 2022-March 2024).

Tumour site	Target type	DL architecture	Additional Info	Dataset (*n*: size)	Imaging modalities	Performance
Brain						
Glioblastoma^23^	GTV	nn U-Net, Swin UNETR	Data Augmentation via registration and GANs. Ensemble of CNN and transformer-based networks.	BraTS 2023 (*n* = 2040)	MRI sequences (T1, T1Gd, T2 and FLAIR)	0.90 DSC 14.94 mm 95th HD
Brain metastases^24^	GTV	GHR-CNN: 2D gated high-resolution CNN	2D architecture. Gated channel attention mechanism	Multi-centre dataset 2012–2022 (*n* = 1592)	MRI (T1c)	0.90 DSC
Head and neck						
Oropharynx^25^	GTV	Auto3DSeg	Model ensemble. Semantic segmentation network with deep supervision.	HECKTOR 2022 (*n* = 883)	CT, FDG PET	0.80 DSC
Lymph nodes^25^	GTV	Auto3DSeg	Model ensemble. Semantic segmentation network with deep supervision.	HECKTOR 2022 (*n* = 883)	CT, FDG PET	0.77 DSC
Nasopharynx^26^	GTV	nn U-Net	Augmentation-invariant framework using intensity transformation strategies.	Multi-centre dataset (*n* = 1057)	MRI (T1)	0.88 DSC 4.99 mm 95th HD
Thorax						
Breast^27^	CTVp	3D U-Net	Multiple U-Net submodels are trained on one or a subgroup of ROIs.	Multi-centre dataset (*n* = 190/200)	CT	0.92-0.94 DSC 8.01-9.38 mm 95th HD
Lung^28^	GTV	2D U-Net with Exponential Linear Unit activations	Multi steps approach: (1) CT data harmonization, (2) lung isolation, (3) tumour detection, (4) tumour segmentation in the detected area	Multi-centre dataset (*n* = 1328)	CT	0.82 DSC 9.43 mm 95th HD
Oesophagus^29^	GTV	3D nn U-Net	Training performed on patches. Sliding window during inference.	Multi-centre dataset (*n* = 580)	CT with contrast	0.87 DSC 2.88-4.33 mm 95th HD
Abdomen						
Kidney^30^	GTV	Auto3DSeg	Bounding box detection for kidneys. Model ensemble from SegResNet and DiNTS algorithms.	KiTS 2023 (*n* = 599)	CT	0.84 DSC
Pancreas^31^	GTV	TD-Net: Trans-Deformer	2D Unet for coarse segmentation and Vision Transformer for fine segmentation.	Multi-centre dataset (*n* = 363)	CT with contrast	0.90-0.91 DSC
Liver^32^	GTV	Three paths encoder-decoder	Multi-scale selective feature fusion, multi-channel feature fusion, edge-inspiring, and edge-guiding modules.	LiTs2017, 3Dircadb (*n* = 151)	CT	0.86 DSC 11.21 mm 95th HD
Pelvis						
Prostate^33^	Prostate gland	2D U-Net	Deep supervision stage in the decoder blocks. Cyclical learning rate.	Multi-centre dataset (*n* = 243)	MRI (T2W)	0.88 DSC
Rectum^34^	GTV CTV	DpnUnet	Two models were trained: one on MRI, GTV as ground truth; one on CT, CTV as ground truth. Model: a U-shape encoder-decoder, locally integrated with dual-path-network modules.	Single-centre dataset (*n* = 141)	MRI CT	0.87 DSC 4.07 mm 95th HD 0.85 DSC 7.75 mm 95th HD
Cervix^35^	CTV	VB-Net	A bottleneck structure replaced the convolution, normalization, and activation layers in V-Net to increase model efficiency.	Single-centre dataset (*n* = 535)	CT	0.86-0.88 DSC

Abbreviations: DL = deep learning; GTV = gross tumour volume; DSC = dice similarity coefficient; 95th HD = Hausdorff distance 95th percentile; CTV = clinical target volume; UNETR = UNet Transformer; GHR-CNN = gated high-resolution convolutional neural network; ROIs = regions of interest; FLAIR = Fluid-attenuated inversion recovery; FDG = Fluorodeoxyglucose.

Compared to the findings of the previously cited review paper^19^, the improvement in performance metrics is evident. For the tumour sites appearing in both reviews, the DSC score is of 0.90 vs. 0.89 for glioblastoma, 0.90 vs. 0.75 for brain metastases, 0.80 vs. 0.76 for oropharynx, 0.82 vs. 0.82 for lung, 0.87 vs. 0.79 for oesophagus, 0.84 vs. 0.85 for kidney, 0.90 vs. 0.73 for pancreas, 0.86 vs. 0.84 for liver, 0.88 vs. 0.94 for prostate, 0.87 vs. 0.86 for cervix. Despite not seeming significantly higher for every tumour site, this improvement is particularly noteworthy given the larger and more heterogeneous datasets used in the cited studies (glioblastoma, brain metastases, and lung are above 1000 cases each), indicating promising advancements in the field. GTV segmentation predominates over tumour CTV segmentation, largely due to (1) the fact that GTV definition corresponds directly to visible imaging data, simplifying model training; (2) the higher variety in tumour CTV delineations, which relies on guidelines, clinical factors and margins that are often not directly discernible in imaging, posing additional challenges to DL models; (3) the greater accessibility of GTV data in openly available datasets. While architectures based on convolutional neural networks (CNN) maintain their dominance over transformer-based ones in most scenarios, significant emphasis is placed on refining pre-processing techniques, employing data augmentation strategies, and leveraging model ensembling, whether with identical or diverse architectures. CT images continue to be the preferred modality across various tumour sites, closely followed by MRI. Unlike CT, MRI protocols tend to exhibit considerable variation across different centres^22^, making the generalization of MRI-based models more challenging. Notably, in this overview, head and neck cancer stands as the sole tumour site reliant on multimodality PET/CT imaging for accurate GTV auto-segmentation, likely due to the availability of easily accessible open multimodality datasets for this specific tumour site.

The images used as input for the models in Table 1 are all high-quality diagnostic scans that facilitate the tumour contouring process. None of the studies included imaging from online adaptive radiotherapy, such as cone-beam CT, likely due to the lower quality of these modalities. Given the limited number of studies focused on these specific imaging types and the fact that only a few tumour sites are treated with online adaptive radiotherapy, we considered them outside the scope of this review. Future research could address the unique challenges associated with using DL models for these modalities and tasks.

## Clinical implementation

**Figure 3.**
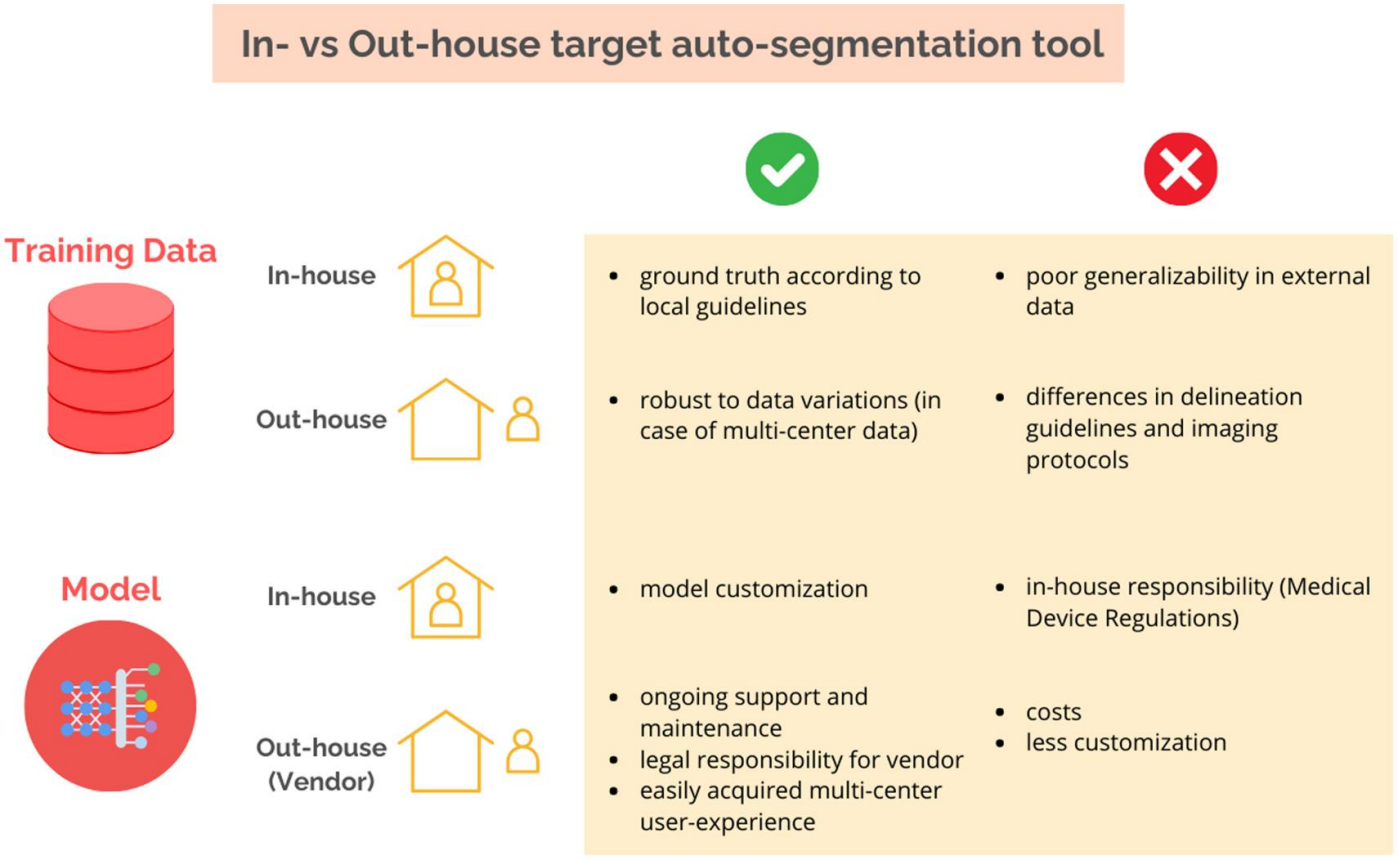
Advantages and disadvantages of using in-house versus out-house data or models in clinical implementation.

Integrating a DL model for target auto-segmentation into clinical practice defines its clinical implementation. Although the literature often distinguishes between in-house and commercially available solutions, differentiating between them can be challenging due to factors such as the data used for training and the models themselves, both of which can be either in-house or out-house. Figure 3 provides an overview of the various possibilities. In this context, an in-house solution refers to the implementation of a model developed internally and trained on institutional data. In-house software for target auto-segmentation offers several advantages. For instance, the ground truth data used for training aligns with local delineation guidelines, likely resulting in fewer adjustments to the outputs and a better understanding of when the model will work well and when it might not. Moreover, in-house solutions are typically developed within hospitals, where data availability is less constrained compared to external companies. However, there are also notable challenges with in-house solutions, particularly related to legal regulations. Both commercial and in-house auto-segmentation tools must comply with medical device regulations (MDR), but while regulatory compliance is handled by vendors for commercial tools, this responsibility falls on the hospital for in-house solutions. This requirement introduces an additional layer of complexity, necessitating careful navigation to ensure compliance and in-house expertise for maintenance and support.

To address some of these challenges, recent studies have utilized internal data to train models provided by external companies. For example, a study by Bakx et al^36^ illustrates this approach in the context of CTV delineation for breast cancer. In their study, the authors trained a DL segmentation model provided by a commercial software company using their patient data. The generated CTV contours were then checked and corrected by radiation oncologists. The entire process of loading scans, checking, and correcting was timed and compared against the time needed for manual segmentation. The study found that radiation oncologists deemed 83% of the CTVs as clinically acceptable or usable as starting point. The results indicate that while some corrections were necessary, the DL approach significantly reduced the time required for segmentation and produced final contours of higher quality compared to the inter-observer variability found in similar studies.

Currently, the commercially available AI solutions for target auto-segmentation are limited to CTVs of a few tumour sites: the prostate^37–42^, the breast^43^, and the head and neck lymph nodes levels for elective irradiation^38^. Notably, all identified studies exclusively utilized single-modality CT. In some studies, the algorithms of these AI solutions were not exposed to the study cohort on which they were tested^38^^,^^41^^,^^42^, while in other cases, the models were re-trained using a small centre-specific dataset^37^^,^^39^^,^^40^. To evaluate the results of AI-driven solutions, the auto-segmented structures are typically compared to manually delineated structures using commonly employed metrics. Notably, in almost all these studies^37^^,^^39–43^, radiation oncologists were also invited to assess the clinical acceptability of the auto-segmentations. In all referenced studies except for^41^, the evaluation was blinded to avoid bias. The criteria for clinical acceptability varied across studies, with some focusing on specific anatomical thresholds for deviations^39^, while others assessed the extent of required corrections^37^^,^^40–43^. Most studies employed a Likert scale for qualitative assessment, though the scale’s granularity varied: some used a 5-point scale^37^^,^^39^, others a 4-point scale^40^, and one used a 3-point scale^43^. The AI auto-segmentations were considered clinically acceptable for treatment planning under the supervision of radiation oncologists who could perform manual editing in outlier scenarios. The studies vary in expert involvement for evaluating auto-segmentations. Studies such as^37^^,^^40^^,^^42^^,^^43^ utilized multiple experts, including panels of radiation oncologists and residents, to ensure a comprehensive assessment. In contrast, Duan et al^39^ and Palazzo et al^41^ relied on evaluations by individual experts, focusing on personal assessments of clinical acceptability and performance.

Why are AI solutions for target auto-segmentation only available for these tumour sites? Firstly, it is crucial to emphasize that prostate gland segmentation benefits from reduced anatomical site variation, as the prostate is an inherent part of anatomy rather than pathology. The same applies to the breast and head and neck elective lymph node levels, as the CTV in these cases does not include the pathologic nodes, but rather the anatomically defined whole breast or head and neck lymph node levels, respectively. Hence, the availability of AI solutions for auto-segmentation of OARs made these targets logical first candidates for target auto-segmentation in clinical practice. Notably, checking and manually editing auto-segmented structures within anatomical regions is significantly more straightforward than doing so within pathological regions, where consultation of multiple imaging modalities is often required.

**Figure 4.**
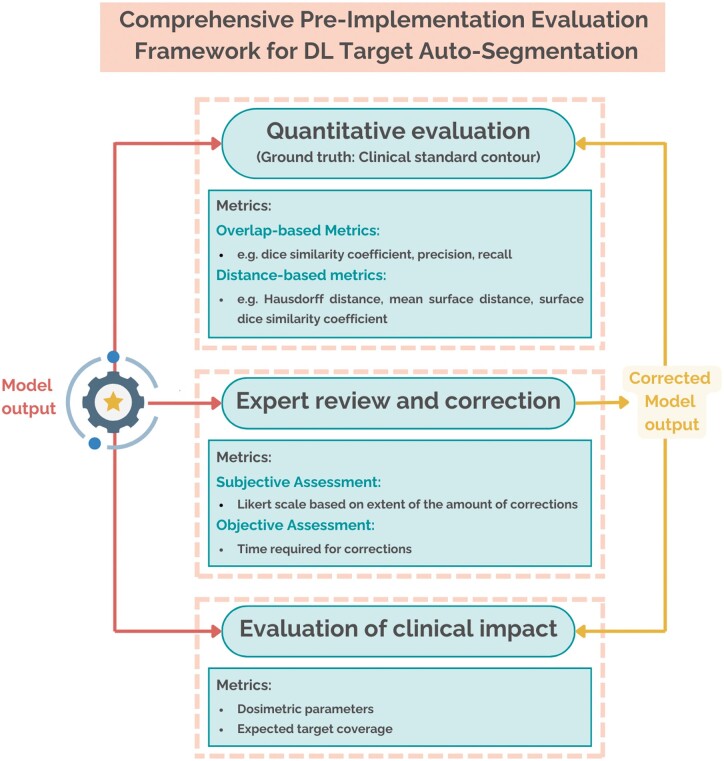
Framework for comprehensive evaluation of DL target auto-segmentation pre-implementation. The evaluation of a DL model’s output for target auto-segmentation should include (1) a quantitative assessment, (2) expert review and correction, and (3) evaluation of the expected target coverage. Abbreviation: DL = deep learning.

What makes the implementation of target auto-segmentation so challenging in clinical practice? One of the foremost challenges still remains offering a *comprehensive performance evaluation* that incorporates both qualitative and quantitative metrics^40^. Currently, in most studies, auto-segmentation results are compared to manually performed contours performed by 1 or 2 radiation oncologists, which are considered the reference. In some cases, however, consensus contours are used, either provided directly by a larger group of experts or generated by combining several individual contours^44^. While this approach offers a more reliable benchmark, it is also more labour-intensive. This highlights the potential need for high-quality, open-source gold-standard datasets that could streamline algorithm validation and provide a robust foundation for consistent evaluation across studies. Overlapping metrics (DSC, precision, recall) are the most commonly used in the majority of technical studies, followed by distance-based metrics (Hausdorff distance, mean surface distance, surface DSC). Those metrics allow an easier and faster comparison of multiple trained DL models, especially during hyperparameter tuning; however, they do not always correlate with clinical acceptability^20^^,^^45^. In addition, in clinical practice, there are more factors to consider in order to prefer automatic systems over manual. It is necessary to prove that the new (or corrected) auto-segmentations allow a full coverage of the target (including dosimetric performance^21^) and ensure time-saving^45^. In a recent study from Ferreira Silvério et al^46^, the authors provided such a comprehensive evaluation when assessing the performance and clinical usability of DL CTV auto-contouring in rectal cancer. They built and tested a framework in which they (1) quantitatively evaluated model performance both against the reference contours and the inter-observer variability; (2) performed expert review and correction of the auto-segmentations, quantifying the time required; (3) evaluated the impact on expected target coverage. In Figure 4, an adapted version of such a framework is provided, summarizing the key aspects for the evaluation of DL target auto-segmentation models.

According to Mackay et al^47^, the *absence of a standardized approach for validating* auto-contouring systems is the root cause of the delay in clinical implementation. When the final goal is to demonstrate that auto-contouring systems can achieve human-level performance, it is crucial to ensure that fair comparisons are conducted across studies. In their review, they provided a multi-step solution consisting of 4 stages: model training and choice, commissioning (physics), commissioning (clinical), case-specific QA. In the first 2 stages, they suggested to produce the ground truth data to use for training, using guidelines and protocols. Additionally, they recommended using commonly employed evaluation metrics to select the best-performing model. The third stage involved a more comprehensive clinical evaluation of the auto-segmentation models, wherein a subset of cases is assessed. This evaluation included comparing the results with intra- and inter-observer variability, as well as evaluating dosimetry and time efficiency. Finally, ongoing QA was proposed as an essential component, involving continuous assessment of validity through quality checks conducted by clinicians for each case.

It is essential to consider broader guidelines or frameworks for the evaluation and continuous QA of auto-segmentation software in radiotherapy. A unified approach endorsed by international organizations could significantly enhance the consistency and reliability of these tools in clinical practice.

## Risk analysis

When introducing a new technology into clinical workflows, it is extremely important to understand its associated risks. In a study from Nealon et al^48^, the authors utilized the failure mode and effects analysis approach to quantify the risks associated with an automated contouring and treatment planning tool which was under development in their institute. Interestingly, among the 290 identified failure modes, the largest group regarded the contour edits, review, and upload process steps in the workflow. This analysis serves as an exemplary model, demonstrating how clinics could approach risk assessments when implementing automated software.

**Figure 5.**
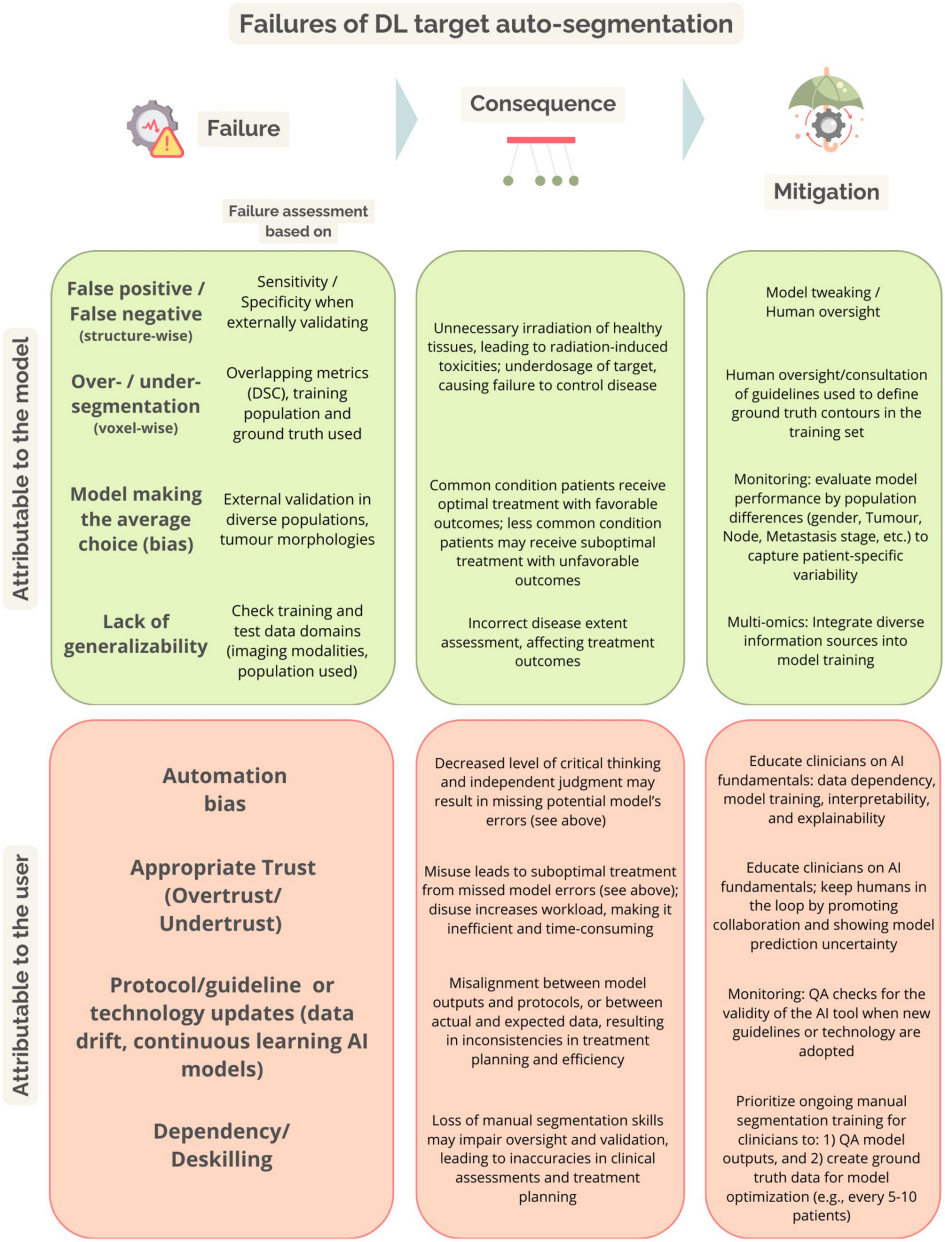
Overview of possible failures attributable to a DL target auto-segmentation model (in green) or to the user (in orange) of a DL target auto-segmentation model. Abbreviation: DL = deep learning.

Based on the findings from Nealon et al^48^ and our own experience, we identified examples of potential failures specifically associated with the clinical use of DL target auto-segmentation models, potential consequences in the radiotherapy treatment setting and potential mitigation strategies. These failures can be attributed to the segmentation model itself or to the end user. Failures of target auto-segmentation models arise from several factors, including the type and quality of the input data, as well as the definition of the ground truth used for model training. Regarding the input data, factors such as image quality, resolution disparities, and variations in tumour or patient characteristics can introduce inconsistencies that challenge segmentation algorithms. Additionally, the presence of artefacts within imaging data can distort segmentation results, compromising the accurate delineation of the target structures. Furthermore, the complexity of defining target boundaries on scans, the segmentation guidelines inclined to clinical interpretation, and the lack of an adequate education can lead to subjective interpretations and discrepancies in segmentations^49^. Inconsistent segmentations used for model training may affect the quality of the model’s output. This challenge is particularly pronounced in tumour sites where target segmentation involves integrating information from multiple imaging modalities and clinical factors, thus adding additional layers of complexity. Relying solely on clinical judgement to establish ground truth introduces subjectivity, as interpretations of a patient’s condition may vary among practitioners. Addressing failures in auto-segmentation requires a comprehensive understanding of the diverse factors influencing both input data type/quality and the definition of a ground truth. Moreover, to mitigate associated clinical consequences and ensure patient safety, it is necessary to have information about the training data, the model performance in external sets, and those populations’ characteristics (see Figure 5).

User interaction and utilization of models for target auto-segmentation may also lead to failures in clinical practice^48^. The clinical risks associated with these failures lie in the trust in target auto-segmentation models, in the potential loss of skills resulting from the adoption of such tools, and the need for continuous updates to align with evolving guidelines and protocols (seeFigure 5). Relying excessively (overtrust) on DL models may result in a decreased level of critical thinking in assessing output target contours, potentially resulting in suboptimal treatment planning and patient outcomes. Conversely, insufficient trust (undertrust) may be an obstacle for technological progress, compromising the transition to innovative techniques like online adaptive radiotherapy where rapid target segmentation is crucial. The involvement of radiation oncologists and radiation therapists is pivotal to ensure that these applications facilitate personalized and high-quality treatment for patients^50^. However, it is necessary that professionals adapt their tasks alongside the technology. In the context of target auto-segmentation, professionals will need to master skills related to data dependency (input imaging and ground truths) and model training, explainability, and interpretability^51^. It is expected, in fact, that a higher experience with AI will lead to a more appropriate use of^52^. Finally, to ensure the accuracy and reliability of the target auto-segmentation model outputs over time, these skills need to be incorporated into robust QA practices.

## Quality assurance

In radiotherapy, many tasks are prone to error that can have significant consequences for patients’ treatment outcomes. Thus, QA procedures are essential standards of care to detect and prevent these potential failures. Human inspection, particularly in the form of peer review, plays a crucial role in traditional manual target segmentation QA^53^. A recent study showed that 38.5% of cases needed changes in head and neck cancer PTV definition after peer review volume rounds. Minor changes were recommended in 16% of cases, whereas major changes were advised in 22.5% of cases. Despite the often subjective nature of minor revisions, the study illustrates that manual contours could benefit from additional editing or consultations before treatment planning^54^. With the introduction of DL target auto-segmentation, it becomes important to routinely monitor DL systems to notice decreased performance due to shifts in protocols, guidelines, technology, or input data distribution. When guidelines change, the solution provided by the system may become outdated for the end user.

Claessens et al^55^ provided a comprehensive overview of QA techniques for AI-based applications in radiotherapy. They delved into auto-segmentation and distinguished between *case-specific QA*, which involves assessing the quality of auto-segmentation on a case-by-case basis, and *routine QA*, which entails periodic monitoring of the model’s output to ensure its overall quality and validity. In *case-specific QA*, human oversight remains indispensable for mitigating the potential associated clinical risks^27^. A common practice involves visual checking and slice-by-slice editing of the output of the auto-segmentation model^56^, which, however, reduces the time-saving of the automated versus the manual segmentation. Considering the complexities of the target segmentation task, largely discussed in the first section, which often require the integration of multiple imaging modalities and careful consideration of patient data, it becomes apparent that manually adjusting auto-segmented target structures can be significantly more time-consuming and challenging compared to OARs.

To speed up the revision process, automated segmentation QA and intuitive human-machine interfaces can enhance time-saving and consistency in contouring. Statistical models are commonly used to detect errors in automatically segmented structures by identifying outliers. These outliers are segmentations that deviate from expected population-based characteristics such as volume, shape, and intensity^57^. These models rely on pre-defined statistical thresholds to find anomalies. Machine learning models are increasingly used to assess segmentation quality. These models are trained to classify whether a segmentation is accurate or not, using the same features but modelling more complex variations in the data^58^. Recently, the topic of uncertainty estimation and quantification drew considerable attention due to its potential dual benefits: (1) reducing the editing/checking time of performed contours by employing metrics that identify cases/areas for which the models are more uncertain in giving predictions; (2) enhancing clinicians’ trust in AI-based auto-segmentation models, enabling a better understanding of where and why these uncertainties arise^19^. In the subsection ‘Towards optimizing case-specific QA: uncertainty estimation’, several solutions for uncertainty estimation will be discussed.

For *routine QA* and *monitoring*, benchmark datasets have been proposed to periodically test end-to-end performance^55^. It is crucial that the datasets accurately represent the real-world patient population^59^, requiring an ongoing upgrade that regularly includes new patients, especially when new scans or guidelines for target segmentation are adopted. The frequency of monitoring should be determined based on these changes and considering potential clinical risks^60^. An alternative monitoring strategy involves conducting statistical analysis on data acquired after manual adjustments have been performed to auto-segmentations. In a recent paper by Nealon et al^61^, the authors showed that tracking the magnitude of required edits over time can help identifying failures attributable to both the model and the end user. An increase in editing could signal deficiencies in the model, while a decrease might imply the end user’s over-reliance on automation.

### Towards optimizing case-specific QA: uncertainty estimation

To ensure that metrics derived from DL estimated uncertainty are suitable for case-specific QA, they must demonstrate well-calibrated relations with segmentation accuracy metrics^62^. Recent literature offers limited studies addressing this topic in target auto-segmentation for radiotherapy. In Table 2, we provide an overview of these studies for various tumour sites, including head and neck^63^^,^^64^, lung^65^, prostate^66^, cervical, and rectal cancer^67^. While promising calibration was generally observed, most studies introduced new metrics to quantify both the estimated uncertainty and segmentation quality, underscoring the absence of a valid and standardized approach. The meaning and implications of uncertainty information for end users remain unclear. Therefore, some studies conducted additional investigations to shed light on its significance. Interestingly, one study^65^ compared DL network uncertainty with areas of uncertainty identified by 2 radiation oncologists, revealing high overall agreement between the 2. In a different work^63^, authors investigated potential correlations between patient-specific characteristics (tumour volume, imaging features) and both segmentation accuracy and uncertainty metrics, showing similar results for the uncertainty in both internal and external test sets.

**Table 2.** Overview of studies on quality assurance using DL uncertainty estimation.

Tumour site (Target)	Imaging modalities	Training dataset (*n*: # test cases)	Uncertainty estimation	Details	Calibration performance
Oropharynx (GTVp)^64^	CT, FDG PET	HECKTOR 2021 dataset (External MDA: *n* = 67)	Deep Ensemble and the Monte Carlo Dropout Ensemble	Segmentation accuracy: DSC Uncertainty quantification metrics: CV, structure expected entropy, structure predictive entropy, and structure mutual information, Dice-risk Calibration quantified by AvU metric and linear correlation between uncertainty and DSC	AvU for CV (both approaches): 0.87 Pearson correlation coefficients (both approaches): 0.72 CV
Oropharynx (GTVp and GTVln)^63^	CT, FDG PET	Internal dataset (Internal: *n* = 104, External (HECKTOR 2022): *n* = 519)	Deep Ensemble of not binarized sigmoid outputs	Output type: Probability maps obtained as the average of multiple outputs of models trained to perform multi-label segmentation. Segmentation accuracy: average of surface DSC calculated after thresholding using different probability thresholds, per patient and per structure. Uncertainty quantification metric: patient and structure-specific CV Calibration: linear correlation between structure-specific CV and average surface DSC	Pearson correlation coefficients: Internal test −0.63 GTVp −0.63 GTVln External test: −0.54 GTVp −0.66 GTVln
Lung (GTV)^65^	CT	Lung1 dataset (*n* = 42)	Monte Carlo dropout with softmax output	Output type: (1) Probability maps calculated as the average of the outputs; (2) Uncertainty maps obtained by normalized entropy Uncertainty quantification metric: MU, RUV Calibration: reliability diagrams Clinical evaluation: correlation between MU and RUV and clinician’s uncertainty estimation (high, intermediate, low uncertainty).	AUC of proposed metrics: 0.849-0.876 MU 0.719-0.804 RUV Pearson correlation coefficients: 0.68 MU 0.49 RUV
Prostate (CTV)^66^	MRI	Multicentre dataset (External: *n* = 49)	Monte Carlo dropout	Output type: (1) the average segmentation of the multiple predictions; (2) a binary uncertainty band obtained thresholding the normalized standard deviation of the predictions Quality assessment: logistic regression that classified manual delineations (pass/discrepancy) based on their spatial association with both the network’s outputs.	AUC = 0.92 TPR = 0.92 FPR = 0.09
Cervix (GTV)^67^	MRI (T2w)	Internal dataset (*n* = 53)	Not binarized softmax output	Output type: ‘score map’ Segmentation accuracy: DSC, 95th HD, MSD, surface DSC Uncertainty quantification metrics: HiS metric = mean of the score map values higher than a threshold (λ = 0.3). Calibration: correlation between HiS and accuracy metrics	Pearson correlation coefficients: 0.79 DSC −0.60 95th HD −0.66 MSD 0.67 surface DSC
Rectal (CTV)^67^	MRI (T2w)	Internal dataset (*n* = 38)			Pearson correlation coefficients: 0.76 DSC −0.53 95th HD −0.73 MSD 0.62 surface DSC

Abbreviations: AvU = accuracy versus uncertainty; CV = coefficient of variation; MU = mean uncertainty; RUV = relative uncertainty volume; AUC = area under the ROC curve; TPR = true positive rate; FPR = false positive rate; DSC = dice similarity coefficient; 95th HD = Hausdorff distance 95th percentile; MSD = mean surface distance.

## Future directions

Will DL target auto-segmentation software ever be independent of human oversight? With the implementation of the new regulations outlined in the EU AI Act^68^, it is unlikely. High-risk AI systems, such as tumour auto-segmentation software, should be designed and developed in such a way that it can be regulated by humans. In line with the risks identified in this review, human oversight remains essential. Thus, research should be carried out to fasten and facilitate the human-machine interaction by proposing solutions that are interactive and efficient, and by combining clinically relevant metrics and visual guidance assessment.

In this context, addressing the challenge of limited annotated data becomes crucial. One promising approach is the Segment Anything Model (SAM)^69^, which has transformed the landscape of image segmentation. SAM generates accurate object masks from natural images using a Vision Transformer-based encoder, a prompt encoder for user interactions, and a mask decoder for reconstructing segmentation masks. Users guide the segmentation process with prompts such as points or bounding boxes around the target. Recently, a new refined version was introduced that is fine-tuned on over 1 million medical image-mask pairs, called MedSAM^70^. Despite the technical progress, research should investigate if and how this type of interactive model can be useful and relevant to radiation oncologists to perform tumour segmentation in clinical practice.

Uncertainty and probability maps represent a promising advancement in facilitating the clinical implementation of target auto-segmentation. Patient-specific QA could be facilitated if adaptive techniques, such as interactive probability maps, would be used for target auto-segmentation. The visual aspect of these maps, combined with well-calibrated uncertainty quantification metrics, would introduce a dynamic element where users are actively engaged, mitigating the risk of over-reliance on automated processes. Recently, a study engaged clinicians in developing a user graphical interface facilitating interaction between radiation oncologists and probability maps derived from a DL GTVp auto-segmentation model trained on PET/CT images of head and neck cancer patients^71^. A user test was also conducted involving 9 expert radiation oncologists to assess the clinical utility of the output. Based on the analysis, end users perceived probability maps as more intuitive and transparent compared to the single contour outputs typically generated by auto-segmentation models. Furthermore, visualizing the model’s confidence in its predictions facilitated a clearer comprehension of the decision-making process underlying the model. An immediate next step involves leveraging the insights from this study for the deployment of similar tools. Subsequently, investigating the potential benefits of such an approach in reducing time requirements and mitigating inter-observer variability is imperative.

In the interaction with the output of DL auto-segmentation models, users should not only consider the model’s segmentation uncertainty (which is ideally informative of segmentation accuracy) but should have a focus on regions where differences in contouring have a larger impact on the patient treatment outcome. In a recent study from Roberfroid et al^72^, the authors proposed the DIVE (Dosimetrically Informed Volume Edition) map, a ‘heatmap’ tool to visually guide clinicians in correcting auto-segmented contours, by only focusing on dosimetrically relevant regions. The tool was evaluated on auto-segmented bladder and rectum from 3 prostate cancer patients treated with adaptive radiotherapy. The preliminary results demonstrated that the tool was able to discard corrections with no dosimetric impact, although its applicability in scenarios different than adaptive radiotherapy may be more challenging to test.

Finally, a reflection is needed on the availability and validity of the ground truth data used to train DL target auto-segmentation models. While manual contours offer the best estimation of tumours based on clinical knowledge and imaging, they do not represent the true ground truth, the histopathological tumour does. Considering that DL models rely on these contours, this raises the question: do manual contours created for radiotherapy treatment accurately represent the histopathological tumour? Only a few studies in literature investigated this topic due to the complexity and uncertainty in aligning imaging with pathological images. Nevertheless, a recent study by Terzidis et al^73^ revealed that PET/MRI imaging tends to overestimate the pathological tumour volume in head and neck squamous cell carcinoma. Improving manual contouring through clearer and more specific guidelines, as well as investigating the relation between imaging and pathology, could improve the consistency of the ground truth data leading to more robust auto-segmentation models.

## Conclusion

The clinical adoption of DL target auto-segmentation is facing delays due to the numerous challenges and considerations it poses. There is a notable discrepancy in the pace at which commercially available software and AI researchers deliver solutions for target auto-segmentation in radiotherapy. In fact, while DL models offer promising solutions to improve efficiency and consistency in target delineation, significant hurdles remain in ensuring their safe and effective utilization. The lack of a standardized approach for QA and monitoring of DL target auto-segmentation models significantly hinders its clinical implementation. Moreover, in the absence of human oversight, DL models may produce target auto-segmentations that could result in suboptimal radiotherapy treatment (over- or under-dosage), with serious consequences for patient outcomes. User interaction with DL target auto-segmentation software may also introduce errors, as a result of overtrust or undertrust, and thereby potentially compromising treatment planning. To mitigate associated clinical risks and ensure patient safety while promoting effective model utilization, strategies include educating professionals, promoting collaborative decision-making, prioritizing ongoing training in manual segmentation skills, and developing robust QA practices. These practices should incorporate human oversight guided by well-calibrated uncertainty quantification metrics, adaptation of professional tasks alongside technological advancements, and ongoing monitoring for end-to-end performance assessment when data evolves. Additionally, further research should delve into expanding the scope of QA to include geometric, dose-volume, and outcome-based performance monitoring. Overall, while DL target auto-segmentation holds great potential to revolutionize radiotherapy treatment planning, careful consideration of clinical risks and rigorous QA measures are imperative for its successful integration into clinical practice.
